# Elosulfase alfa for mucopolysaccharidosis type IVA: Real-world experience in 7 patients from the Spanish Morquio-A early access program

**DOI:** 10.1016/j.ymgmr.2018.03.009

**Published:** 2018-04-05

**Authors:** Guillem Pintos-Morell, Javier Blasco-Alonso, María L. Couce, Luís G. Gutiérrez-Solana, Encarna Guillén-Navarro, Mar O'Callaghan, Mireia del Toro

**Affiliations:** a Department of Pediatrics, University Hospital and Research Institute Germans Trias i Pujol, Badalona, Universitat Autònoma de Barcelona, Spain; b Unidad de Gastroenterología, Hepatología y Nutrición Infantil, Hospital Regional Universitario de Málaga, Spain; c Unit of Diagnosis and Treatment of Congenital Metabolic Diseases, Service of Neonatology, Department of Pediatrics, Hospital Clínico Universitario de Santiago, CIBERER, University of Santiago de Compostela, Health Research Institute of Santiago de Compostela (IDIS), Santiago de Compostela, Spain; d Section of Neuropediatrics, Hospital Infantil Universitario Niño Jesús, Madrid, Spain; e Consejería de Sanidad, IMIB-Arrixaca, Murcia, Spain. Grupo Clínico CIBERER-ISCIII, Spain; f Department of Neuropediatrics, Hospital Sant Joan de Déu, Esplugues, Barcelona, Spain; g Pediatric Neurology Unit, Hospital Universitari Vall d'Hebron, Barcelona, Spain

**Keywords:** MPS IVA, Morquio A, Lysosomal storage disorder, Urinary GAGs, Elosulfase alfa, Quality of life

## Abstract

There is a growing interest in evaluating the effectiveness of enzyme replacement therapy (ERT) with elosulfase alfa in patients with mucopolysaccharidosis type IVA (MPS-IVA) under real-world conditions. We present the experience of seven pediatric MPS-IVA patients from the Spanish Morquio-A Early Access Program. Efficacy was evaluated based on the distance walked in the 6-min walking test (6-MWT) and the 3-min-stair-climb-test (3-MSCT) at baseline and after 8 months of ERT treatment. Additionally, urinary glycosaminoglycans were measured, and a molecular analysis of a GALNS mutation was performed. The health-related quality of life was evaluated using the EuroQoL (EQ)-5D-5 L.

The distance walked according to the 6-MWT ranged from 0 to 325 m at baseline and increased to 12–300 m after 8 months with elosulfase alfa (the walked distance improved in all patients except one). An increase was observed for the two patients who had to use a wheelchair. Improvements were also observed for the 3-MSCT in four patients, whereas two patients showed no changes. Three patients showed an improvement in the EQ-VAS score, whereas the scores of three patients remained stable. Regarding urinary glycosaminoglycans measurements, an irregular response was observed. Our results showed overall improvement in endurance and functionality after 8 months of elosulfase alfa treatment in a heterogeneous subset of MPS IVA patients with severe clinical manifestations managed in a real-world setting.

## 1 Introduction

Mucopolysaccharidosis type IVA (MPS IVA; Morquio A syndrome, OMIM 253000) is an autosomal recessive lysosomal storage disorder (LSD) that was first described by Luis Morquio and James Brailsford in 1929 [1]. MPS IVA is caused by a deficiency of the *N*-acetylgalactosamine-6-sulfate sulfatase (GALNS) enzyme, which leads to a progressive accumulation of the glycosaminoglycans (GAGs) chondroitin-6-sulfate (C6S) and keratan sulfate (KS). The accumulation of undegraded C6S and KS triggers progressive systemic skeletal dysplasia in MPS IVA patients [[2], [3], [4], [5]]. Significant non-skeletal manifestations, including respiratory disease, spinal cord compression, cardiac disease, impaired vision, hearing loss, and dental problems, have also been described in these patients [[6], [7], [8]]. MPS IVA is more frequently associated with severe and extensive skeletal manifestations than the other MPS types. Specifically, hypermobility of the joints is a characteristic of MPS IVA that distinguishes this disease from the other types. Furthermore, these patients exhibit no cognitive involvement [9]. In the mild form, the symptoms can appear as late as the second decade of life [6], and some patients can reach a normal stature [[10], [11], [12]]. In contrast, patients with a severe form of MPS IVA may not survive beyond the second or third decade of life.

The recent availability of enzyme replacement therapy (ERT) with recombinant human GALNS (elosulfase alfa; Vimizin®; BioMarin Europe Ltd., London, UK) has provided systemic treatment for MPS IVA when added to traditional symptom-based management. Elosulfase alfa was shown to be effective and had an acceptable tolerability profile in a phase 1/2, open-label, dose-escalation study and a pivotal 24-week, randomized, double-blind, placebo-controlled clinical trial [13,14], which led to marketing authorization for elosulfase alfa as a treatment for MPS IVA by FDA in February 2014 and by the European Medicines Agency in April 2014. Nevertheless, patients with MPS IVA have very heterogeneous clinical phenotypes due to the multiple systems affected by the disorder, and no studies have evaluated how this therapy works in a real-world setting. The present report describes our experience using elosulfase alfa treatment of seven pediatric patients from the Spanish Morquio A Early Access Program (MOR-EAP).

## 2 Materials and methods

The MOR-EAP started in 2014 as a compassionate use program to provide access to elosulfase alfa for patients with MPS IVA after approval from the European Medicines Agency (April 28, 2014) while they waited for inclusion in the Spanish public health system. The patient inclusion criteria were similar to the criteria described for the pivotal clinical trial [14]. Individuals had a confirmed diagnosis of MPS IVA as documented by GALNS molecular genetic testing. The exclusion criteria included prior hematopoietic stem cell transplantation (HSCT) or a concurrent disease or condition that would interfere with ERT. The MOR-EAP was undertaken according to the Spanish regulations on access to medicines under special conditions [15]. Written informed consent was obtained from the parents or a legally authorized representative together with the child's assent.

All patients included in the program received elosulfase alfa (2.0 mg/kg/week) and were pre-treated with an antihistamine drug to reduce the risk of infusion-related adverse reactions. During the program, the following efficacy information was collected: the distance walked in the 6-MWT [16] and the 3-MSCT [17]. Additionally, respiratory function tests were performed according to published European Respiratory Society and Spanish Pediatric Pneumology Association guidelines [18], including the forced vital capacity (FVC), forced expiratory volume in one second (FEV1) and FEV1/FVC ratio. The clinical examinations also included anthropometric measurements (weight, length and body mass index [BMI]), audiometry measurements, vital signs, electrocardiograms and echocardiograms, an ophthalmological examination, magnetic resonance imaging of the brain and spine, and radiographs of the lower extremities and the spine.

Total urinary GAGs were measured according to an improved dimethylene blue (DMB) test. This method involved assaying GAGs in the DMB dye followed by a spectrophotometric analysis of the resulting complex. The normal values for this method depend on age [19]. Molecular analysis of GALNS mutations was performed as previously described [20].

The health-related quality of life (HRQoL) was measured with the EuroQoL (EQ)-5D-5L questionnaire [21]. We use the EQ-5D-5L questionnaire, as recommended by the international guidelines for the management and treatment of MPS IVA [22]. This questionnaire is a simple but complete self-administered measure of the HRQoL for a wide range of health conditions that has been translated into 106 languages [21]. This generic standardized tool comprises five dimensions (mobility, self-care, usual activities, pain/discomfort and anxiety/depression). The results are presented as the EQ Visual Analog Scale (VAS) score as a measure of the overall self-rated health status, for which the data range from 100 (the best state one can imagine) to 0 (the worst state one can imagine). Adverse reactions were also recorded during the 8-month period.

## 3 Results

**Fig. 1:**
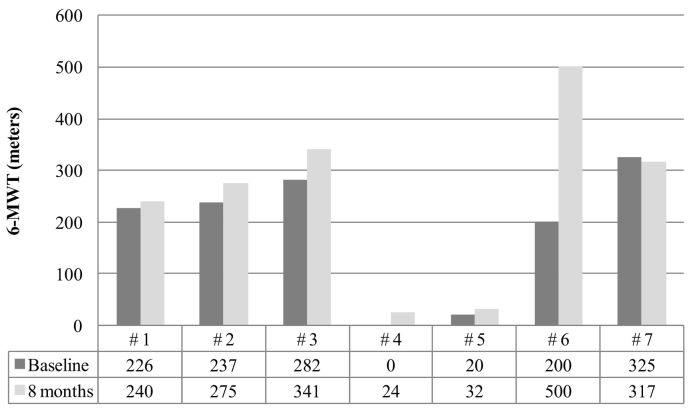
Assessment of endurance according to the 6-min walking test (6-MWT) at baseline and after 8 months of elosulfase alfa therapy in the Morquio A Early Access Program (MOR-EAP). A 0 score was given to patients who did not walk.

**Fig. 2:**
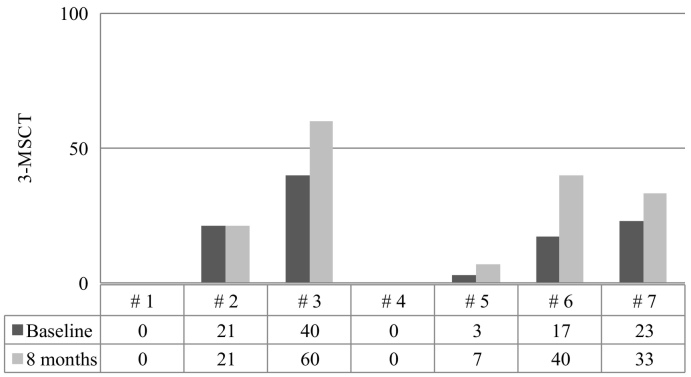
Assessment of endurance according to the 3-min-stair-climb-test (3-MSCT) at baseline and after 8 months of elosulfase alfa therapy in the Morquio A Early Access Program (MOR-EAP). 3-MSCT: Results are given as stairs/min.

This early access program included seven patients aged 7–17 years. The clinical features of these patients before they began elosulfase alfa treatment are presented in Table 1. The evolution of endurance was evaluated with the 6-MWT and the 3-MSCT, which are represented in Fig. 1, Fig. 2, respectively. An improvement in the walking distance (6-MWT) was observed after 8 months of treatment with elosulfase alfa in all but one patient (Fig. 1).

**Table 1:** Anthropometric, clinical and health-related quality of life assessments of patients at baseline and after 8 months of elosulfase alfa therapy in the MorquioA Early Access Program (MOR-EAP).

	Patient number													
	#1		#2		#3		#4		#5		#6		#7	
	7 years old		7 years old		7 years old		11 years old		13 years old		16 years old		17 years old	
	Baseline	8 months	Baseline	8 months	Baseline	8 months	Baseline	8 months	Baseline	8 months	Baseline	8 months	Baseline	8 months
Weight (Kg)	16.5	16.8	16.0	17.2	22.0	24.0	19.4	24.0	31.4	31.8	35.2	35.6	48.3	NA
Lenght (cm)	95.0	96.0	91.4	93.0	120.0	124.0	107.0	108.0	113.0	113.8	145.5	145.5	137.0	137.0
BMI	18.3	18.2	19.2	19.9	15.2	15.6	16.9	20.5	24.6	24.5	16.6	16.9	25.7	NA
FVC (%)	63.0	75.0	85.3	92.0	95.0	95.0	86.0	75.0	67.0	74.0	100.0	104.0	123.0	132.0
FEV1 (%)	60.0	50.0	75.6	82.0	94.0	93.0	93.0	80.0	70.0	83.0	99.0	102.0	127.0	135.0
Echocardiogram	Mit In +, AO	NA	Mit In +, AO	Mit In +, AO	Normal	Normal	IVSHT+	Normal IVS	MIT IN +, IVS	MIT IN+	MIT IN+, AO	MIT IN+, AO	MIT IN+	MIT IN+
MRI	Mult spinal	Mult spinal	C1-C3	C1-C3	C1-C2	C1-C2	C1-C2	C1-C2	C2	C2	Cervical	Cervical	Stenosis D11	Stenosis D11
Corneal opacity	+	+	+	+	Normal	Normal	+	+	+	+	Normal	Normal	+	+
Hypoacusia	+	+	++	++	Normal	Normal	++	++	+	++	+	+	+	+
EQ-5D-5 L, EQ VAS score	50	65	25	45	50	50	50	50	45	45	NA	NA	60	75
GALNS mutation	p.G301C/T394P [c.901G > T] + [1180A > C]		p.G47R/p.P781LfsX9		p.R386C/[A231G/H238H] + E477E		p.R386C/p.G301C		p.G301C I R386H [c.901G > T] + [c.1157G > A]		p.Glu121Asp (c.363G > C) Homozigosis		p.[G301C + W141X] /c. [901G > T + 423-566del]	
Urine GAGs (mg/mmol creatinine)	NA	NA	17.9	NA	23.0	<limit	19.8	7.4	9.0	12.0	5.7	3.8	1.7	NA

Abbreviations: BMI, body mass index; EQ-5D-5L, EuroQoL 5 dimensions; FEV, forced expiratory volume (% predicted); FVC, forced vital capacity (% predicted); GAGs, glycosaminoglycans; Mit In: Mitral Insufficiency; Ao In: Aortic Insufficiency; IVS HT: Interventricular Septum Hypertrophy; MRI, magnetic resonance imaging; Mult spinal, multiple spinal abnormalities; NA, not available.

Results for the EQ-5D-5 L are given as EQ VAS score, which ranged from 100 (the best state one can imagine) to0 (the worst state one can imagine).

Improvements were also observed in the 3-MSCT in four subjects; whereas two subjects showed no change and one subject had no data available (Fig. 2). A marked improvement was observed in patient #6, whose scores nearly doubled on both tests. Patients #4 and #5 were not independently mobile and had to use a wheelchair (baseline 6-MWT distances of 0 and 20 m, respectively). However, these patients also showed improvement in their walking distances after 8 months on elosulfase alfa (6-MWT distances of 24 and 32 m, respectively; Fig. 1).

Information on the anthropometric characteristics, clinical assessments, urine GAG levels and HRQoL evaluation results at baseline and after 8 months of ERT is shown in Table 1. Four patients had urine GAGs measured at baseline and at 8 months; of those patients, three showed a reduction in GAGs at the end of the follow-up period (Table 1). During this period, small variations in respiratory function were observed after 8 months of treatment with elosulfase alfa (Table 1). Thus, five patients showed improvements in their FVC ranging from a 4% to 19% increase from baseline, one patient had an unchanged FVC and another patient had a reduced FVC. The cardiac function evaluation showed no changes with the exception of two patients who showed normalization of their intraventricular septum hypertrophy. The magnetic resonance imaging (MRI) findings also remained unchanged, and similar results were obtained for the hearing and ophthalmologic evaluations. The evolution of the quality of life as measured with the EQ-5D-5 L VAS score is presented in Table 1. Patients #1, #2 and #7 showed an improvement in their HRQoL scores after 8 months of treatment, whereas three patients remained stable. The HRQoL was not evaluated in one patient. In the pre-pubertal patients (<9 and 10 years old for girls and boys, respectively), the range of growth was 0.8 to 4 cm. The elosulfase alfa infusion was well tolerated, and no serious adverse events were reported. No treatment disruption or surgical intervention was required during the study.

## 4 Discussion

This early access program in a group of highly heterogeneous patients with MPS IVA showed that elosulfase alfa provided benefits to the patients in terms of endurance and functionality in a real-world setting that were similar to the benefits reported in the randomized pivotal trial [23].

The data available from the 7 MOR-EAP patients at baseline support the findings in natural history studies that describe this rare disease as a progressive, multi-organ/systemic, heterogeneous pathology [6,7,[24], [25], [26]]. Most of the cases described in the present study showed not only a variety of clinical manifestations in the musculoskeletal system (several forms of stenosis –segmental and multisegmental-, dysplasia and myelopathy) but also breathing difficulties, cardiac valve disease, impaired vision (corneal opacity) and hearing loss. Several factors may have determined the severity of the disease, and although no definition has been currently proposed, growth and final height are commonly accepted as indicators of disease severity in MPS IVA [27,28]. Patients in this case series were classified as severe MPS IVA with the exception of patient #3, who had a less severe manifestation of the disease. Overall, we believe that this report may be of interest to clinicians who need information in addition to the data from explanatory randomized trials regarding the effects of elosulfase alfa therapy under real-world conditions.

The 6-MWT and 3-MSCT results revealed an important impairment of endurance and mobility in this MPS IVA population regardless of age. The distance walked according to the 6-MWT ranged from 0 to 325 m at baseline. The lower limit for healthy individuals 4–16 years of age ranges from 470 to 664 m [29,30]. Several studies have shown similar scores at baseline [7,[24], [25], [26]], but the natural course of the disease involves decreasing endurance as patients age [22]. The administration of elosulfase alfa to patients with MPS IVA in this 8-month program led to improvements in walking distances in all but one patient. The improvements ranged from 12 to 300 m, and in three patients the improvement was above the mean increase of 37 m reported in the pivotal trial [23]. Interestingly, two patients who showed a walking distance in the 6-MWT below 30 m (patients #4 and #5), which was an exclusion criterion in the pivotal trial for elosulfase alfa [23], also experienced improvements in walking distance (24 and 12 m). Based on an exploratory subgroup analysis, we hypothesized that elosulfase alfa treatment might be more effective in patients with more severe disease at baseline (i.e., in patients who could walk a distance ≤200 m) [23,31]. The results of this case series do not support that hypothesis, because the changes in the walking distance in three of the five subjects who walked >200 m at baseline were above the walking distance in that subgroup from that exploratory analysis (38, 59 and 300 m vs. a mean of 25 m). In contrast to the pivotal trial, which found a trivial improvement in the 3-MSCT (i.e., +1.1 stairs/min) [23], we found a relevant improvement in the 3-MSCT in four patients (three patients had an increase of >10 stairs/min), including one patient who was unable to walk a distance >30 m in the 6-MWT but exhibited an increase of 4 stairs/min. The results of the 3-MSCT were remarkable, especially considering that studies hypothesized that this test might not be suitable for patients with MPS IVA because these patients had severe skeletal dysplasia, short stature and joint involvement that considerably limited their ability to climb stairs [26]. Patient #7, who showed a slight decrease in the walking distance in the 6-MWT, experienced an improvement in the 3-MSCT. This lack of correlation between the 6-MWT and 3-MSCT results was also found in the pivotal trial with elosulfase alfa, in which the performance in the 6-MWT did not translate to better performance in the 3-MSCT [23,31]. The 6MWT measures the integrated function of at least 3 separate organ systems that are affected by MPS IV A: the respiratory, cardiovascular, and musculoskeletal systems. The mechanism of improvement is not well established but could be related to the improvement of respiratory function and pain.

Reduced respiratory function was also observed in our series at baseline with limited volumes in FVC for all the MOR-EAP individuals. Treatment with elosulfase alfa led to a slight improvement in respiratory function for most patients. These results were also consistent with the results of the pivotal trial, which showed a slight increase in the FVC and forced expiratory volume in 1 s (FEV1) after a follow-up period of 24 months [23]. However, the observation period in our series may have been too short to evaluate the impact of elosulfase alfa on respiratory functions, because the extended study period of the pivotal trial with elosulfase alfa showed that the percentage change from baseline in the FVC increased from <4% at 24 weeks to 7–8% at 72 and 120 weeks [32]. In healthy children, the longitudinal improvement in pulmonary function was found to be age-dependent, with the greatest increase occurring during puberty [26]. Height increase and thoracic enlargement most likely facilitate respiratory functions and therefore increase the FVC and MVV volumes in patients<14 years old [26]. However, the older patients in our series (#6 and #7), reached their highest FVC and FEV volumes. The more impaired respiratory functionality was observed in patient #1, who was only 7 years old. Nevertheless, individuals #2 and #3 were also 7 years old, and their FVC and FEV1 volumes were greater and similar to each other.

Cardiovascular involvement in MPS IVA patients can lead to early mortality. Thus, rapid intervention may be life-saving [33]. We did not find relevant changes in the echocardiograms after elosulfase alfa therapy. Similarly, the ophthalmological and audiometric evaluations were unchanged under ERT at 8 months. The results of urinary GAGs were difficult to analyze. On one hand, not all patients with MPS IVA had elevated GAG levels at diagnosis. On the other hand, the levels may decrease naturally with age, which may explain the low baseline levels found in patients #6 and #7 [22]. Almost all studies use this measure, but no data are available to support its usefulness in the clinical management of MPS IVA [34]. It is possible that measurement of urinary keratan sulfate instead of total GAGs, and a more homogeneous population would give more consistent results.

For a debilitating disease such as MPS IVA, evaluating a patient's HRQoL is of paramount importance. Reduced endurance or mobility, difficulties in activities of daily living, dependence on caregivers, frequent surgical interventions, pain and fatigue are factors that are frequently reported by patients with MPS IVA [7,8]. The global EQ-VAS scores ranged from 45 to 60 at baseline with the exception of patient #2, who had a more severe condition. However, this patient reported a clinically meaningful improvement after 8 months of elosulfase alfa therapy. Of the 5 remaining evaluated patients, 2 showed improvement in the HRQoL and 3 showed no changes, including the two patients with the most severe mobility problems (patients #4 and #5). There is the possibility of improvement of some clinical variables by motivation or training.

In conclusion, there is a growing interest in the availability of real-world data from patients with rare diseases [35], including MPS IVA [36]. This case series provided a first look into the effectiveness of elosulfase alfa treatment in patients with MPS IVA under real-world conditions. Overall, our results showed improvements in endurance and functionality with elosulfase alfa that were at least similar to the improvements reported in the explanatory setting of the pivotal trial, including the results from two patients who were markedly affected by mobility problems (i.e., patients excluded from the pivotal trial) who exhibited a modest but relevant improvement in endurance and showed no changes in their HRQoL. However, in addition to the limitations inherent to small case series, more information is needed on the impact of elosulfase alfa on other outcomes that are relevant to the patients, such as fatigue, joint stiffness and pain [31]. Therefore, our results should be confirmed with long-term multidimensional data from registries.
